# A Novel Protein Interaction between Nucleotide Binding Domain of Hsp70 and p53 Motif

**DOI:** 10.1155/2015/391293

**Published:** 2015-05-18

**Authors:** Asita Elengoe, Mohammed Abu Naser, Salehhuddin Hamdan

**Affiliations:** Department of Biosciences and Health Sciences, Faculty of Biosciences and Medical Engineering, Universiti Teknologi Malaysia, 81310 Skudai, Johor, Malaysia

## Abstract

Currently, protein interaction of *Homo sapiens* nucleotide binding domain (NBD) of heat shock 70 kDa protein (PDB: 1HJO) with p53 motif remains to be elucidated. The NBD-p53 motif complex enhances the p53 stabilization, thereby increasing the tumor suppression activity in cancer treatment. Therefore, we identified the interaction between NBD and p53 using STRING version 9.1 program. Then, we modeled the three-dimensional structure of p53 motif through homology modeling and determined the binding affinity and stability of NBD-p53 motif complex structure via molecular docking and dynamics (MD) simulation. Human DNA binding domain of p53 motif (SCMGGMNR) retrieved from UniProt (UniProtKB: P04637) was docked with the NBD protein, using the Autodock version 4.2 program. The binding energy and intermolecular energy for the NBD-p53 motif complex were −0.44 Kcal/mol and −9.90 Kcal/mol, respectively. Moreover, RMSD, RMSF, hydrogen bonds, salt bridge, and secondary structure analyses revealed that the NBD protein had a strong bond with p53 motif and the protein-ligand complex was stable. Thus, the current data would be highly encouraging for designing Hsp70 structure based drug in cancer therapy.

## 1. Introduction

p53 gene acts as a tumor suppressor in many tumor cells. It is a labile protein which is comprised of 393 amino acids, folded and unstructured regions that function in a synergistic manner [1] and responsible for induction of cell cycle arrest or apoptosis based on the cellular damage, stress, and cell type. In cell cycle, it acts as a transactivator that negatively regulates cell division by controlling a set of genes required for this process such as cyclin-dependent kinases, protein kinases, and cyclin-dependent inhibitors. In apoptosis, it is responsible for highly complex and sophisticated processes involving energy-dependent cascade of molecular events [2]. Induction of apoptosis is mediated either by BAX stimulation and antigen expression of FAS or by repression of Bcl-2 expression. However, p53 gene modification could cause uncontrolled cell proliferation.

Human Hsp70 functions as ATP-dependent molecular chaperone which plays a crucial role in proteins folding, assembly or disassembly of other protein structures, and protecting from cell stress [3, 4]. Human Hsp70 has 640 amino acids and is comprised of two structurally independent domains such as nucleotide binding domain (NBD) and C-terminal substrate binding domain (SBD). NBD (residues 1–380) is responsible for ATPase activity while SBD (residues 397–641) plays the key role in binding of peptides and folding of nonnative polypeptides. In addition, a hydrophobic linker of 13 residues (384–396) that carries a highly conserved leucine-rich motif (EEVD) is connected between NBD and SBD [5].

There has been evidence that the* Xenopus laevis* p53 can bind to mammalian Hsp70 protein [6]. The formation of p53-Hsp70 complex might enhance stabilization of p53 in cancer cells, thus increasing killing efficiency of cancer cells. However, the process of Hsp70 molecular chaperone involved in the buffering of p53 conformation and activity is not clear up until now.

In this study, we have explored for the first time how the NBD interacts with p53 motif. Three-dimensional structure of p53 motif was constructed through homology modeling. We then determined the binding affinity and stability of NBD-p53 motif complex structure through molecular docking and dynamics (MD) simulation. Therefore, the results could help in designing the structure based drug Hsp70 for cancer treatment.

## 2. Materials and Methods

### 2.1. Identification of Protein Interaction between HSPA1A and p53

STRING version 9.1 program is used to find the interaction between HSPA1A and p53 [7].

### 2.2. Target Sequence

The three-dimensional structure of HSPA1A is publicly available. The complete amino acid sequence of HSPA1A was obtained from RCSB Protein Databank (PDB: 1HJO). It consists of 380 amino acids. The protein model was visualized using PyMol software [8].

### 2.3. Homology Modeling

Currently, there is no tertiary structure of* Homo sapiens* DNA binding domain of p53 available in the protein database. The complete amino acid sequence of DNA binding domain of p53 was retrieved from UniProt (UniProtKB: P04637). BLASTP against the RCSB Protein Databank was carried out to find a suitable template for homology modeling. Crystal structure of 1YCS was selected as a template based on maximum identity with high positives and lower gaps percentage. The percentages of query coverage, sequence identity, positivity, and gap between the template and target protein were 100%, 100%, 100%, and 0%, respectively. The three-dimensional structure of DNA binding domain of p53 was built using EasyModeller 2.1 software [9], the graphical user interface (GUI) of Modeller 9.10 [10], and the model was then viewed using PyMol software. The three-dimensional model of the DNA binding domain of p53 motif (SCMGGMNR) was created using the built three-dimensional model of DNA binding domain of p53 as a template. The Gromacs package 4.6.3 [11] adopting the GROMOS 53a6 force field parameter was employed to perform 50 ns MD simulation for the p53 motif before blind docking with the NBD protein.

### 2.4. Protein-Protein Docking

The three-dimensional model of NBD protein was used for molecular docking with the tertiary structure of p53 motif (SCMGGMNR) using Autodock version 4.2 program [12]. In the protein, nonpolar hydrogen atoms were merged and total Kollman and Gasteiger charge was added to the protein. It was ensured that there were no unbound atoms in the protein. Kollman and Gasteiger partial charges were also assigned to the ligand and all torsions were allowed to rotate during docking. The NBD and p53 motif were converted from the PDB format to the PDBQT format. A grid box was used around the active site to cover the entire protein binding site and allow ligands to move freely; and affinity maps of NBD (74 × 88 × 108 containing total grid points of 727575) were calculated by AutoGrid. One hundred Lamarckian Genetic Algorithm (LGA) runs with default parameter settings were performed. Docking was reclustered for 0.5, 1.0, and 2.0 tolerances. The largest docked conformations were clustered at RMS of 1.0 nm and played according to the rank of the native Autodock scoring function. The best conformation with the lowest docked energy was chosen from the docking search. The interactions of complex NBD protein-p53 motif conformations including hydrogen bonds and bond lengths were analyzed.

### 2.5. Molecular Dynamics Simulation of the NBD-p53 Motif Complex

In the 50 ns MD simulation, the docked complex of p53 motif with NBD protein was simulated using the GROMACS package 4.6.3 adopting the GROMOS53a6 force field parameter [11]. The protein topology was constructed by pdb2gmx with GROMOS53a6 force field. We used a cubic box setting a minimal distance of 1.0 between the protein and edge of the box, which was then solvated using periodic boundary conditions and the SPC (simple point charge) water model in this study. The ligand topology file was generated using the PRODRG server to include the heteroatom due to limitations of GROMACS to parameterize the heteroatom group in the PDB file [13]. To make the system neutral, we added one sodium ion around the molecule for the NBD. The entire system for the NBD protein was minimized using 993 steps of steepest descent, respectively. After energy minimization with particle-mesh Ewald algorithm at every step, the system was then equilibrated at a constant temperature (303 K), volume, number of particles in system, and pressure (1 bar) for 50 ps. Under constant volume equilibration, the temperature was maintained by Berendsen weak coupling method. Moreover, under constant pressure equilibration, the temperature was controlled by Berendsen weak coupling method and the pressure was maintained by Parrinello-Rahman barostat method. After completion of the two equilibration phases, production of MD simulation was conducted for 1 ns after taking away the position restraints. Finally, the equilibrated structures were subjected to MD simulations for 50 ns (50,000 ps) with linear constrain (LINCS) algorithm 2 fs time step to constrain all the bonds. The nonbonded list was generated using an atom-based cut-off of 10 Å. The trajectory snapshots were taken for structural analysis at every picosecond. Root mean square deviations (RMSD), backbone atomic fluctuations (RMSF), secondary structure, hydrogen bonds, and salt bridge between the protein and ligand in the docked complex during the simulation were analyzed through Gromacs utilities g_rmsd, g_rmsf, do_dssp, g_hbond, and g_salt, respectively.

## 3. Results and Discussion

### 3.1. Identification of Protein Interaction between HSPA1A and p53

**Figure 1:**
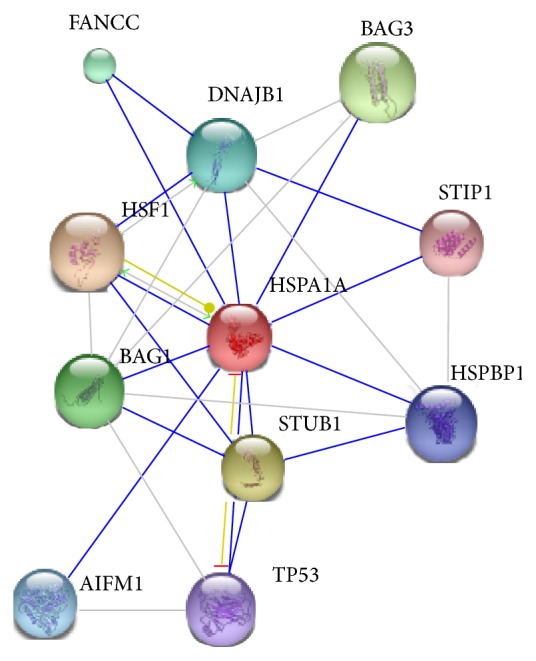
Protein interaction of HSPA1A with p53 was found through STRING version 9.1 program.

*Homo sapiens* HSPA1A-p53 interaction was identified using STRING version 9.1 program.  Figure 1 shows that HSPA1A either directly interacted with p53 or used intermediate STUB1 to connect with p53. STUB1 is STIP1 homology and U-box containing protein 1 which modulates several chaperone complexes' activity such as Hsp70, Hsc70, and Hsp90. It has E3 ubiquitin-protein ligase activity and targets misfolded chaperone substrates towards proteasomal degradation. It also mediates transfer of noncanonical short ubiquitin chains to HSPA8 that have no effect on degradation of HSPA8. The binding score for interaction of HSPA1A-p53 was 0.908. According to STRING, only two experimental studies demonstrated the detection of human HSPA1A-p53 complex by Affinity Capture Western assay and* in vivo* assay.

In mammalian cancer lines, HSPA1A was found to associate with p53 in the cytoplasm but the complex dissociates upon translocation of p53 into the nucleus. Both of the experimental results show that p53-Hsp70 complex formation might be one of the mechanisms of stabilization of p53 protein resulting in its increased levels in potentially malignant and malignant tumors [14, 15]. However, to date, the process of Hsp70 molecular chaperone participating in the buffering of p53 conformation and activity is not known clearly, where p53 plays important role in inhibition of tumorigenesis.

Based on studies by Fourie et al. (1997), Rüdiger et al. (2002), and Müller et al. (2004), it has been described that Hsp70 interacts with p53 DBD (DNA binding domain) [16–18]. p53 DBD contains 191 amino acids (from residue 102 to residue 292). Mutations usually occur in DBD. This causes deactivation of p53 by destroying the ability of the protein to bind to its target DNA sequences, thus preventing transcriptional activation of these genes. This type of mutation is defined as recessive loss-of-function mutations. For example, mutation of p53 DBD at residue 248 (arginine) distorts the protein structure. There are three regions in p53 DBD such as region 1 (from residue 113 to 226) which is required for interaction with FBX042; region 2 (from residue 116 to 292) involved in interaction with AX1N1; and region 3 (from residue 241 to 248) that interacts with the 53BP2 SH3 domain. SCMGGMNR (region 3) transmits antiproliferative signals through 53BP2 SH3 domain to antiapoptotic Bcl2 family proteins (Bcl-2, Bcl-W, and Bcl-XL). Therefore, molecular dynamics simulation and docking approaches have been used to determine the preferred sites, binding affinity, and stability of the HSPA1A-p53 motif complex structure.

### 3.2. Protein-Protein Docking

**Figure 2:**
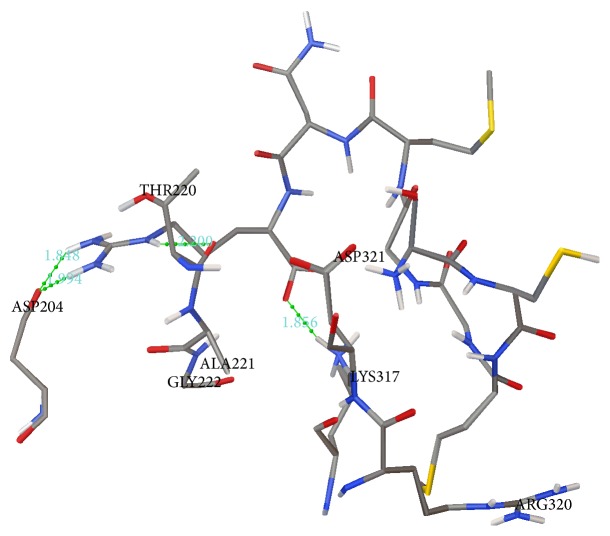
Human DNA binding domain of p53 motif (SCMGGMNR) retrieved from UniProt (UniProtKB: P04637) was docked with the NBD protein, using the Autodock version 4.2 program. Hydrogen bonds are shown by a green line with its distance (Å).

The docking analysis revealed that NBD had a strong bond with p53 motif due to negative and low binding energy (−0.44 Kcal/mol). Thus, the protein was in its most favorable conformation. The total intermolecular energy of NBD-p53 motif complex was −9.9 Kcal/mol. This indicates that the protein model has a good binding affinity with ligand. Other binding parameters of p53 motif such as electrostatic energy (3.70 Kcal/mol), internal energy (−4.23 Kcal/mol), torsion energy (9.55 Kcal/mol), unbounded extended energy (4.23 Kcal/mol), cluster RMS (0.0), and reference RMS (61.53) were also determined. In addition, the NBD protein formed four hydrogen bonds with p53 motif (Figure 2 and Table 1).

### 3.3. Model Simulation and Evaluation of Protein-Ligand Complex

**Figure 3:**
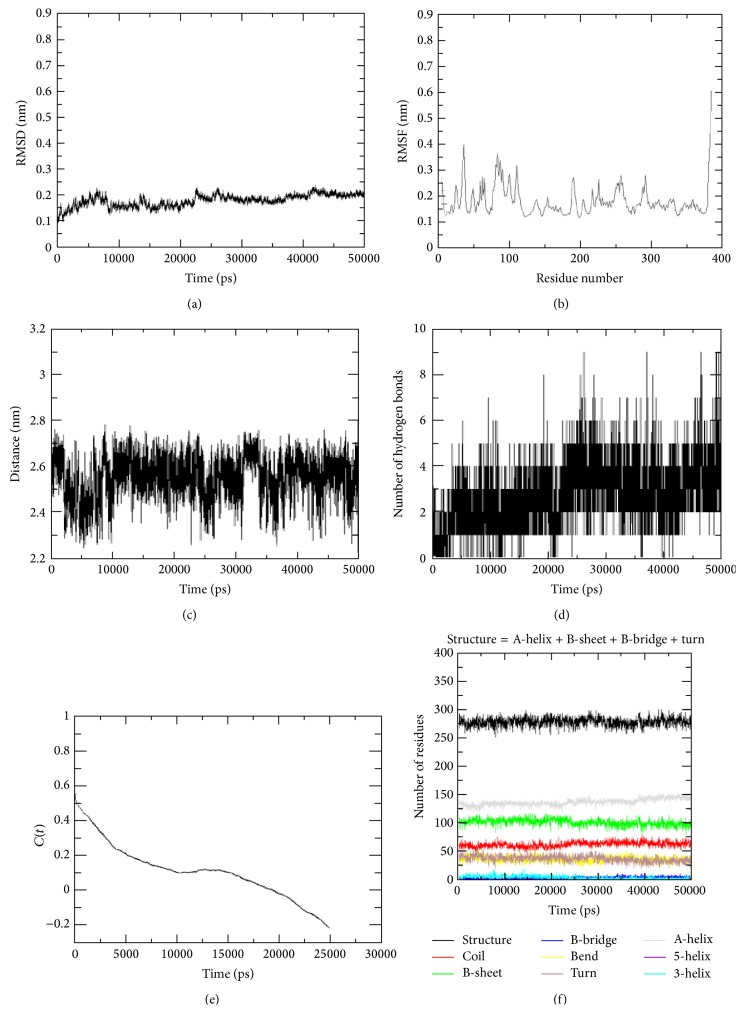
Stability studies of NBD-p53 motif complex structure. (a) Root mean square deviations (RMSD); (b) backbone atomic fluctuations (RMSF); (c) salt bridge; (d) number of hydrogen bonds; (e) hydrogen bond autocorrelation; and (f) secondary structure analysis of the NBD-p53 motif complex during the 50 ns molecular dynamics simulation.

The NBD-p53 motif complex was run for 50 ns molecular dynamics simulation. The RMSD value of the protein-ligand complex model against the simulation period is shown in Figure 3(a). It can be seen that the NBD-p53 motif complex achieved a stable value at 45,000 ps (0.20 nm). Moreover, the RMSD value was less deviated throughout the 50 ns simulation time. Results from RMSF analysis show that all the residues in the protein structure fluctuated between 0.05 and 0.30 nm in the entire simulation period (Figure 3(b)). The NBD-p53 complex exhibited a high fluctuation up to 0.54 nm at residue 380.

Furthermore, salt bridges occurring between the NBD protein and p53 motif are demonstrated in Figure 3(c). The protein-ligand complex exhibited a stable distance of 2.50 nm throughout the simulation period. The deviation in flexibility was further analyzed by the number of hydrogen bonds formed between the NBD protein and p53 motif during MD simulation (Figure 3(d)). The hydrogen bond analysis revealed that NBD-p53 motif complex shows four intermolecular hydrogen bonds, which were determined throughout the simulation period. At least 1-2 intermolecular hydrogen bonds were kept for the entire 50 ns trajectories, which inferred the stability of NBD-p53 motif complex.

In addition, history-independent hydrogen bond autocorrelation function was calculated between the protein and the ligand (Figure 3(e)). The life time of hydrogen bond between protein and ligand is 25,000 ps. This describes that the bond between the protein and ligand is strong. Furthermore, the secondary structure also plays an essential role in stability of such a system.  Figure 3(f) indicates that the alpha-, 5-, and 3-helixes were constant throughout the simulation period which infers that the protein-ligand complex structure was stable.

## 4. Conclusion

RMSD, RMSF, salt bridge, hydrogen bond, and secondary structure analyses have proven that the protein interaction between the NBD protein and p53 motif was stable. In addition, NBD of HSPA1A had a strong bond with p53 motif (SCMGGMNR). Therefore, the current data would be highly encouraging for* in vitro* experiments of gene therapy in cancer treatment. Moreover, a better understanding of* in silico* interpretations of this protein structure will aid in the development of target-specific anticancer drugs for cancer treatment.

## Figures and Tables

**Table 1:** Hydrogen bonds interaction study of the NBD protein with p53 motif (SCMGGMNR).

Protein	Donor atom of protein	Acceptor atom of ligand	Distance (Å)
NBD	ARG8:HE	THR220:O	2.399
	ARG8:HH22	ASP204:OD1	1.848
	LYS317:HZ1	ARG8:O1	1.856
	ARG8:HH21	ASP204:OD1	1.994
